# Pet-Keeping in Relation to Asthma, Rhinitis, and Eczema Symptoms Among Adolescents in Kuwait: A Cross-Sectional Study

**DOI:** 10.3389/fped.2020.00331

**Published:** 2020-06-23

**Authors:** Khadijah A. AlShatti, Ali H. Ziyab

**Affiliations:** ^1^Faculty of Medicine, Kuwait University, Safat, Kuwait; ^2^Department of Community Medicine and Behavioral Sciences, Faculty of Medicine, Kuwait University, Safat, Kuwait

**Keywords:** pets, allergy, asthma, eczema, rhinitis, cat, dog, rabbit

## Abstract

**Background:** Findings on the associations between household pet-keeping and allergic diseases, including asthma, rhinitis, and eczema, have been contradictory, with investigations reporting positive, negative, and null associations. This study sought to estimate the prevalence of pet-keeping among families in Kuwait and to assess the associations between pet-keeping and symptoms of allergic diseases among adolescents.

**Methods:** Schoolchildren aged 11–14 years (*n* = 3,864) were enrolled in this cross-sectional study. The children's parents completed questionnaires regarding their child's environmental exposures, including pet-keeping in the past 12 months, and clinical history and symptoms of allergic diseases. Associations were assessed using Poisson regression with robust variance estimation, and adjusted prevalence ratios (aPRs) and their 95% confidence intervals (CIs) were estimated.

**Results:** Pet-keeping in the past 12 months was reported by 42.8% of the participating families. Birds, cats, rabbits, fish, and dogs were kept by 28.3, 13.2, 7.8, 3.9, and 3.1% of all households, respectively. Current cat ownership was significantly associated with current wheezing (aPR 1.29, 95% CI 1.05–1.58), current rhinitis symptoms (aPR 1.18, 95% CI 1.02–1.36), and ever doctor-diagnosed eczema (aPR 1.25, 95% CI 1.03–1.50). Current rabbit-keeping was positively associated with multiple symptoms of asthma (e.g., study-defined current asthma: aPR 1.38, 95% CI 1.04–1.82) and eczema (e.g., severe eczema: aPR 1.94, 95% CI 1.02–3.71). Similarly, current bird-keeping was associated with study-defined current rhinitis (aPR 1.19, 95% CI 1.05–1.41) and current itchy rash (aPR 1.27, 95% CI 1.10–1.46).

**Conclusions:** Household pet-keeping is very common and diverse in Kuwait and was found to be positively associated with symptoms of allergic diseases among adolescents. The findings of associations between rabbit-keeping and symptoms of asthma and eczema add to the existing literature and further highlight the importance of considering the pet type when assessing such associations.

## Introduction

Asthma, rhinoconjunctivitis, and eczema are chronic allergic diseases affecting both children and adults that are common globally. They impose a substantial burden on public health resources worldwide. According to the International Study of Asthma and Allergies in Childhood (ISAAC), the global average prevalence estimates of current asthma, rhinoconjunctivitis, and eczema symptoms are 14.1% (1), 14.6% (2), and 7.3% (3), respectively, among adolescents aged 13–14 years. Global variations in the prevalence and time trends of allergic diseases were noted in investigations carried out by ISAAC (4, 5). The between- and within-nation disparities suggest that local environmental factors in interaction with genetic elements could explain the observed variations in the prevalence of allergic diseases. A prior ISAAC study conducted among schoolchildren aged 13–14 years in Kuwait estimated the prevalence of symptoms of asthma, rhinitis, and eczema to be 16.1, 30.7, and 12.6%, respectively (6). Such estimates indicate that the burden of allergic diseases among adolescents in Kuwait is comparable to that reported in westernized nations (4).

Several environmental factors have been reported to be associated with allergic diseases among children, such as environmental tobacco smoke, ambient air pollution, climatic factors, viral infections, and indoor and outdoor allergen exposure (7–13). Household pets are a major source of animal allergens, endotoxins, and microbes, which can modulate the risk of sensitization and subsequent allergic diseases among susceptible individuals (14, 15). Thus, far, conflicting findings on the associations between exposure to pets (mainly cats and dogs) and allergic diseases have been reported. Some studies have shown protective effects of pet exposure on allergic disease development, whereas other studies have shown that pet-keeping is a risk factor for allergic diseases. Furthermore, some investigations have reported no associations between pet exposure and allergic diseases (16–22). It has been speculated that the observed protective effect of pet exposure is a consequence of reverse causation due to selective avoidance, wherein allergy-susceptible families may refrain from having pets or parents may give away or avoid having pets after disease onset in their children (18). However, recent studies exploring the role of reverse causation in previously reported associations between environmental factors (including pet-keeping) and wheezing and eczema have provided evidence against reverse causation (selective avoidance), hence strengthening the possibility of a causal link (23, 24). Moreover, analysis based on ISAAC data has indicated that associations of pet exposure with wheezing/eczema are stronger in non-affluent countries, where selective avoidance behavior is less likely (23, 24).

Given that cats and dogs are the most common pets in western/westernized societies, most prior studies have focused on the relationship between cat/dog exposure and allergic diseases. Therefore, the role of other pets (e.g., birds, rabbits, fish, etc.) in allergic diseases is less explored and might provide intriguing results. Moreover, the distribution of pet-keeping could differ across nations or even communities on the basis of social and cultural acceptance. To this end, because of the lack of empirical knowledge, we aimed to report the prevalence of pet-keeping among families with children in Kuwait and to assess the associations between exposure to household pets and symptoms of asthma, rhinitis, and eczema among adolescents.

## Methods

### Study Setting, Design, and Participants

Kuwait is a small country overlooking the Persian (Arabian) Gulf, with a total area of approximately 18,000 km^2^. Geographically, Kuwait is divided into six governorates, and the school districts follow a similar geographic distribution. Education in Kuwait is mainly provided by free public schools funded by the state and, to a lesser extent, by private schools. The education system can be divided into four stages, namely, kindergarten, elementary school (1st−5th grade), middle school (6^th^-9th grade), and high school (10th−12th grade), and in the latter three stages, the students are segregated by sex. Schooling is compulsory for all children aged 6–14 years.

This cross-sectional study enrolled schoolchildren (*n* = 3,864) attending public middle schools throughout the State of Kuwait, which included children aged between 11 and 14 years. The schoolchildren were enrolled in the study during the 2016–2017 school year (September 2016 to May 2017) and the first semester of the 2017–2018 school year (September to December 2017). A stratified two-stage cluster sampling method was used to select a representative study sample of schoolchildren from a random sample of schools across Kuwait. The sampling methodology is described in detail elsewhere (25, 26). Ethical approval for the current study was obtained from the Standing Committee for Coordination of Health and Medical Research, Ministry of Health, Kuwait (no. 2016/451). The study was conducted in accordance with the principles and guidelines of the Declaration of Helsinki for medical research involving human subjects.

The children were asked to take home the study-specific questionnaire and a standardized questionnaire [i.e., the ISAAC questionnaire (27)] for parental/guardian completion and return them to the school authorities. The questionnaires gathered information on demographic data, lifestyle factors, environmental exposures (including pet-keeping), and clinical history and symptoms of allergic diseases among both the children and their parents. Written informed consent for study participation was obtained from each child's parents or legal guardians.

### Ascertainment of Pet Exposure

The current (past 12 months) pet-keeping status was determined by asking the parents the following questions:

1. In the past 12 months, have you had a cat in your home?
2. In the past 12 months, have you had a dog in your home?
3. In the past 12 months, have you had other types of pets in your home (other than a cat or a dog)? [If the parents answered “yes,” they were asked to list the types of pets].

In addition to cats and dogs, the following pets and pet groups were reported, in response to question 3: birds, rabbits, fish, reptiles (turtles and snakes), poultry (domestic chickens, ducks, and pigeons), and rodents (hamsters and squirrels). “Any pet” was defined as the presence of any type of pet at home in the past 12 months.

### Definitions of Asthma, Rhinitis, and Eczema Symptoms

#### Asthma Symptoms

Asthma symptoms were defined according to the ISAAC methodology (1). Current wheezing was ascertained by asking the following question: “Has your child had wheezing or whistling in the chest in the past 12 months?” Having current dry cough at night was determined by an affirmative response to the following question: “In the past 12 months, has your child had a dry cough at night, apart from a cough associated with a cold or chest infection?” Symptoms of severe asthma included the presence of current wheezing and the occurrence of ≥4 wheezing attacks, sleep disturbance from wheezing ≥1 night per week, or wheezing-affected speech in the past 12 months. Ever doctor-diagnosed asthma cases were identified by asking the parent whether the child had ever been diagnosed as having asthma by a doctor. Study-defined current asthma (i.e., asthma in the past 12 months) cases were identified by an affirmative response to the items “history of doctor-diagnosed asthma” and “wheezing in the past 12 months” and/or “asthma treatment in the past 12 months” (25).

#### Rhinitis Symptoms

The following core questions from the ISAAC questionnaire (27) were used to define rhinitis and rhinoconjunctivitis symptoms according to the criteria defined in ISAAC reports (2):

1. In the past 12 months, has your child had a problem with sneezing or a runny or blocked nose, when he or she did not have a cold or the flu?
2. In the past 12 months, has this nose problem been accompanied by itchy-watery eyes?
3. In the past 12 months, how much did this nose problem interfere with your child's daily activities? [Possible answers are: not at all, a little, a moderate amount, a lot].
4. Has your child ever been diagnosed by a doctor with rhinitis?

Question 1 was used to define current (12 month) rhinitis symptoms. Affirmative responses to both questions 1 and 2 were used to ascertain the presence of current rhinoconjunctivitis symptoms. Positive responses to questions 1 and 2 and the answer “a lot” to questions 3 were used to assess the presence of current severe rhinoconjunctivitis symptoms. Moreover, an affirmative response to question 4 was used to determine whether the child had ever been diagnosed as having rhinitis by a doctor. Study-defined current rhinitis was defined as “ever doctor-diagnosed rhinitis” and “having problems with a sneezing, runny, or blocked nose in the absence of a cold or flu in the past 12 months” (25).

#### Eczema Symptoms

Eczema symptoms were defined according to the ISAAC methodology (3). The presence of a current itchy rash was determined by asking the following question: “Has your child had this itchy rash any time in the past 12 months?” Moreover, an affirmative response to the question “Has this itchy rash at any time affected any of the following places: the folds of the elbows, behind the knees, in front of the ankles, under the buttocks, or around the neck, ears or eyes” was used to determine the presence of a current itchy flexural rash. These questions were preceded by the following question: “Has your child ever had an itchy rash coming and going for at least 6 months?” The presence of current severe eczema was defined as having a current flexural rash associated with sleep disturbance ≥1 night per week. The presence of ever doctor-diagnosed eczema was assessed by asking whether the child had ever been diagnosed as having eczema by a doctor. According to the criteria defined by Hanifin and Rajka (28), study-defined current eczema was defined as “ever doctor-diagnosed eczema” and/or “having ever had a recurrent itchy rash for at least 6 months” plus “having a current itchy flexural rash.”

### Covariates

Information regarding exposures and covariates was obtained from questionnaires completed by the parents/guardians. As body mass index (BMI), which is a measure of general adiposity, markedly changes in children with growth, we estimated the BMI-for-age z-scores [standard deviation (SD) scores] using the WHO growth reference for children aged between 5 and 19 years (29). The BMI-for-age score was categorized as follows: underweight (thinness): < -2 SD, normal: −2 to 1 SD, overweight: >1 to 2 SD, and obese: >2 SD (29). Exposure to environmental tobacco smoke (ETS) was assessed by inquiring whether any member of the household smoked cigarettes or tobacco-related products inside the house. Breastfeeding status was determined by asking whether the child was ever directly breastfed during infancy. The questionnaire also asked about the total number of siblings and number of older and younger siblings the child had. Moreover, information on maternal and paternal highest educational attainment was collected. Maternal and paternal history of allergy were defined as ever doctor-diagnosed asthma, rhinitis, and/or eczema.

### Statistical Analysis

All statistical analyses were carried out using SAS 9.4 (SAS Institute, Cary, North Carolina, USA). The statistical significance level was set at α = 0.05 for all association analyses. Descriptive analyses were carried out to determine the frequencies and proportions of categorical variables and the medians and 5th and 95th percentiles of quantitative variables. The current (12-month) prevalences of asthma, rhinoconjunctivitis, and eczema symptoms were estimated, along with their binomial 95% confidence intervals (CIs). Similarly, the prevalence estimates of exposure to pets in the past 12 months were determined.

Adjusted associations were assessed by applying a modified Poisson regression with robust variance estimation using the GENMOD procedure in SAS 9.4 to estimate and infer the adjusted prevalence ratios (aPRs) and their 95% CIs (30). We evaluated the associations of different types of pets (exposure variables) with different symptoms of asthma, rhinoconjunctivitis, and eczema (outcomes variables). The pet variables (i.e., cat, dog, rabbit, bird, poultry, fish, and reptile) were all entered into multivariable regression models, hence simultaneously adjusting their effects. Moreover, all multivariable regression models were adjusted for sex, age, breastfeeding, mode of birth, environmental tobacco smoke exposure, body mass index, maternal history of allergy, paternal history of allergy, maternal education, paternal education, number of older siblings, and number of younger siblings.

## Results

### Characteristics of Study Sample

The total number of schoolchildren participating in the study was 3,864 (response proportion: 73.9%, 3,864/5,228), which included 1,695 (43.9%) boys and 2,169 (56.1%) girls, with a median age of 12 years (Table 1). With regard to BMI-for-age groups, 25.4 and 28.8% of children were overweight and obese, respectively. Of all children, 45.8% reported exposure to household environmental tobacco smoke. The majority of parents reported having a bachelor's degree or higher. Moreover, the prevalence of maternal and paternal history of allergy (i.e., asthma, rhinitis, and/or eczema) was estimated to be 45.4 and 45.1%, respectively (Table 1).

**Table 1:** Characteristics of the total study sample.

**Variable**	**Total study sample (*n* = 3864)**
**Sex**, ***n*** **(%)**	
Male	1,695 (43.9)
Female	2,169 (56.1)
**Age (years)**, Median (5th, 95th percentile)	12.0 (11.0, 14.0)
**BMI-for-age groups**, ***n*** **(%)**	
Underweight (< −2 SD)	219 (5.8)
Normal (−2 to 1 SD)	1,517 (40.0)
Overweight (> 1 to 2 SD)	961 (25.4)
Obese (> 2 SD)	1,089 (28.8)
Missing, *n*	78
**Mode of Birth**, ***n*** **(%)**	
Vaginal	3,106 (81.8)
Cesarean section	692 (18.2)
Missing, n	66
**Breastfeeding ever**, ***n*** **(%)**	
Yes	2,894 (76.3)
Missing, *n*	72
**Number of older siblings**, ***n*** **(%)**	
0	1,103 (28.7)
1	801 (20.8)
2	638 (16.6)
≥ 3	1,302 (33.9)
Missing, n	20
**Number of younger siblings**, ***n*** **(%)**	
0	544 (14.4)
1	757 (20.0)
2	798 (21.2)
≥ 3	1,674 (44.4)
Missing, n	91
**Environmental tobacco smoke exposure**, ***n*** **(%)**	
Yes	1,755 (45.8)
Missing, *n*	28
**Maternal history of allergy**, ***n*** **(%)**	
Yes	1,691 (45.4)
Missing, *n*	142
**Paternal history of allergy**, ***n*** **(%)**	
Yes	1,533 (41.8)
Missing, *n*	195
**Maternal Education**, ***n*** **(%)**	
Middle school or less	556 (14.6)
High school	705 (18.5)
Diploma^*^	929 (24.3)
Bachelor's degree or higher	1,630 (42.7)
Missing, *n*	44
**Paternal Education**, ***n*** **(%)**	
Middle school or less	686 (18.0)
High school	877 (23.0)
Diploma^*^	875 (23.0)
Bachelor's degree or higher	1,360 (35.8)
Missing, n	66

*BMI, body mass index; SD, standard deviation*.

* * Refers to a 2-year associate degree post high school*.

### Symptoms of Asthma, Rhinitis, and Eczema

The prevalence estimates of asthma, rhinitis, and eczema symptoms are shown in Table 2. Current wheezing was reported by 15.7% of study participants and severe asthma symptoms had been experienced by 7.9% of the total study sample. Current symptoms of rhinitis and rhinoconjunctivitis were reported by 28.6 and 13.5% of the study sample, respectively. With regard to eczema symptoms, a current itchy rash and a current itchy flexural rash were reported by 20.5 and 11.3% of study participants, respectively (Table 2).

**Table 2:** Prevalence estimates of asthma, rhinitis, and eczema symptoms.

**Symptoms**	**% (*n*/total)**	**95% CI**
**Asthma**		
Current wheeze	15.7 (573/3,660)	14.5–16.8
Current dry cough at night	38.0 (1,362/3,584)	36.4–39.6
Severe asthma	7.9 (290/3,660)	7.1–8.8
Ever doctor-diagnosed asthma	23.5 (887/3,782)	22.1–24.8
Study-defined current asthma	15.7 (600/3,829)	14.5–16.8
**Rhinitis**		
Current rhinitis symptoms	28.6 (1,040/3,643)	27.1–30.0
Current Rhinoconjunctivitis	13.5 (497/3,689)	12.4–14.6
Severe rhinoconjunctivitis	1.19 (44/3,689)	0.8–1.5
Ever doctor-diagnosed rhinitis	24.9 (927/3,726)	23.5–26.3
Study-defined current rhinitis	15.1 (566/3,759)	13.9–16.2
**Eczema**		
Current itchy rash	20.5 (735/3593)	19.1–21.8
Current itchy flexural rash	11.3 (417/3701)	10.3–12.3
Severe eczema	1.7 (64/3701)	1.3–2.2
Ever doctor-diagnosed eczema	19.5 (736/3775)	18.2–20.8
Study-defined current eczema	10.2 (388/3791)	9.3–11.2

### Pet-Keeping Prevalence

**Figure 1:**
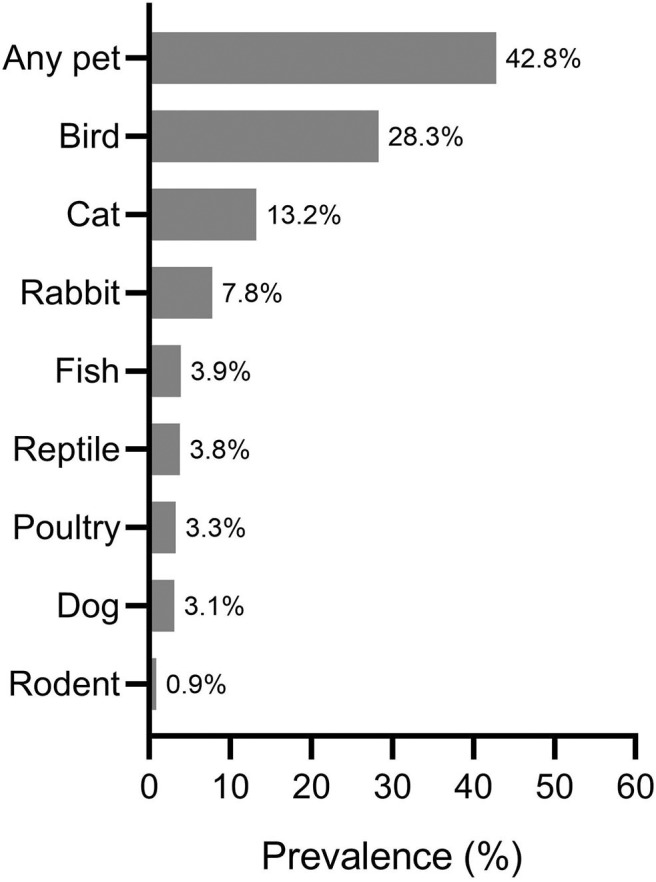
Prevalence estimates of household pet-keeping in the past 12 months. Prevalence estimates are shown according to pet type. Also, prevalence estimate of having “any pet” is shown.

The prevalence estimates of pet-keeping in the past 12 months according to pet types/groups are provided in Figure 1 and online Table S1. The prevalence of any pet-keeping was estimated to be 42.8%. Birds, cats, rabbits, fish, and dogs were kept by 28.3, 13.2, 7.8, 3.9, and 3.1% of all participating families, respectively (Figure 1).

### Parental History of Allergy and Pet-Keeping

Estimates of pet-keeping according to maternal and paternal history of allergy (i.e., asthma, rhinitis, and/or eczema) are shown in Table 3. In general, the prevalence of pet-keeping was higher in families with a maternal or paternal history of allergy. For example, among participants with a maternal history of allergy, 31.8% reported keeping birds compared with 25.6% among those without a maternal history of allergy. Similarly, any pet-keeping was higher in families with a maternal/paternal history of allergy (Table 3).

**Table 3:** Prevalence estimates of pet-keeping according to maternal and paternal history of allergy.

**Pet type**	**Maternal history of allergy**			**Paternal History of allergy**		
	**No, % (*n*/total)**	**Yes, % (*n*/total)**	***P*-value**	**No, % (*n*/total)**	**Yes, % (*n*/total)**	***P*-value**
Cat	12.6 (255/2,019)	14.1 (237/1,680)	0.188	12.6 (267/2,127)	13.8 (209/1,520)	0.290
Dog	2.32 (47/2,025)	4.0 (67/1,682)	0.004	2.7 (57/2,128)	3.5 (53/1,528)	0.168
Bird	25.6 (516/2,014)	31.8 (532/1,672)	<0.001	26.6 (562/2,116)	30.8 (468/1,519)	0.005
Rabbit	7.5 (150/2,014)	8.3 (138/1,672)	0.364	6.9 (145/2,116)	8.6 (130/1,519)	0.055
Poultry	2.8 (57/2,014)	3.8 (64/1,672)	0.091	2.9 (62/2,116)	4.0 (61/1,519)	0.074
Reptile	3.7 (74/2,014)	4.1 (68/1,672)	0.537	3.5 (75/2,116)	4.2 (63/1,519)	0.348
Fish	3.1 (63/2,014)	4.8 (81/1,672	0.007	3.4 (71/2,116)	4.4 (67/1,519)	0.102
Rodent	0.8 (17/2,014)	0.9 (15/1,672)	0.863	1.0 (21/2,116)	0.7 (10/1,519)	0.280
Any pet	39.6 (802/2,027)	46.9 (791/1,687)	<0.001	40.1 (855/2,133)	45.9 (702/1,529)	<0.001

### Associations of Pet-Keeping With Asthma, Rhinitis, and Eczema Symptoms

Table 4 shows the associations between current exposure to household pets and current symptoms of asthma, rhinitis, and eczema. Current cat ownership was significantly associated with current wheezing (aPR 1.29, 95% CI 1.05–1.58), current rhinitis symptoms (aPR 1.18, 95% CI 1.02–1.36), and ever doctor-diagnosed eczema (aPR 1.25, 95% CI 1.03–1.50). In this study, there were no statistically significant associations between keeping a dog and current symptoms of asthma, rhinitis, or eczema (Table 4). Statistically significant associations were found between rabbit exposure and asthma symptoms, including current nocturnal cough (aPR 1.18, 95% CI 1.02–1.37), ever doctor-diagnosed asthma (aPR 1.30, 95% CI 1.04–1.62), and study-defined current asthma (aPR 1.38, 95% CI 1.04–1.82). In addition, rabbit-keeping was associated with severe eczema symptoms (aPR 1.94, 95% CI 1.02–3.71) and study-defined current eczema (aPR 1.45, 95% CI 1.07–1.20). Furthermore, bird-keeping was associated with ever doctor-diagnosed rhinitis (aPR 1.21, 95% CI 1.07–1.36), study-defined current rhinitis (aPR 1.19, 95% CI 1.05–1.41), and current itchy rash (aPR 1.27, 95% CI 1.10–1.46). Reptile-keeping demonstrated an association with reporting current nocturnal cough (aPR 1.22, 95% CI 1.01–1.48; Table 4).

**Table 4:** Associations between pet type/group and asthma, rhinitis, and eczema symptoms.

**Symptoms**	Adjusted PR^*^ **(95% CI)**						
	**Cat**	**Dog**	**Rabbit**	**Bird**	**Poultry**	**Fish**	**Reptile**
**Asthma**							
Current wheeze	**1.29 (1.05–1.58)**	0.95 (0.64–1.41)	1.22 (0.91–1.65)	1.01 (0.85–1.20)	0.92 (0.57–1.46)	0.85 (0.54–1.34)	0.73 (0.45–1.18)
Current dry cough at night	1.00 (0.88–1.14)	1.12 (0.91–1.38)	**1.18 (1.02–1.37)**	1.06 (0.96–1.16)	1.13 (0.93–1.39)	1.03 (0.84–1.26)	**1.22 (1.01–1.48)**
Severe asthma	1.18 (0.86–1.62)	0.59 (0.27–1.27)	1.42 (0.94–2.16)	1.07 (0.83–1.39)	0.40 (0.15–1.05)	1.32 (0.74–2.36)	0.50 (0.22–1.16)
Ever doctor-diagnosed asthma	1.15 (0.98–1.36)	1.18 (0.90–1.55)	**1.30 (1.04–1.62)**	0.93 (0.82–1.07)	0.93 (0.66–1.33)	1.21 (0.91–1.61)	0.76 (0.53–1.08)
Study-defined current asthma	1.16 (0.94–1.43)	1.28 (0.90–1.80)	**1.38 (1.04–1.82)**	0.96 (0.82–1.14)	0.88 (0.55–1.40)	1.06 (0.71–1.58)	0.79 (0.50–1.24)
**Rhinitis**							
Current rhinitis symptoms	**1.18 (1.02–1.36)**	0.99 (0.74–1.31)	1.15 (0.95–1.40)	1.02 (0.91–1.14)	1.00 (0.75–1.34)	0.85 (0.63–1.15)	0.81 (0.59–1.12)
Current Rhinoconjunctivitis	1.21 (0.96–1.53)	0.97 (0.63–1.50)	1.02 (0.74–1.41)	1.04 (0.86–1.24)	0.94 (0.58–1.52)	0.80 (0.49–1.28)	0.89 (0.55–1.43)
Severe rhinoconjunctivitis	0.47 (0.15–1.51)	1.66 (0.42–6.56)	1.30 (0.47–3.61)	1.57 (0.84–2.95)	–^†^	–^†^	–^†^
Ever doctor-diagnosed rhinitis	1.00 (0.84–1.18)	1.00 (0.74–1.36)	1.15 (0.93–1.41)	**1.21 (1.07–1.36)**	0.85 (0.60–1.21)	0.73 (0.52–1.02)	0.91 (0.66–1.26)
Study-defined current rhinitis	1.05 (0.84–1.32)	0.85 (0.54–1.32)	1.20 (0.90–1.61)	**1.19 (1.05–1.41)**	1.00 (0.65–1.55)	0.69 (0.42–1.12)	0.62 (0.36–1.05)
**Eczema**							
Current itchy rash	1.14 (0.95–1.38)	0.71 (0.46–1.10)	1.24 (0.98–1.56)	**1.27 (1.10–1.46)**	0.98 (0.69–1.40)	0.97 (0.68–1.37)	1.15 (0.83–1.59)
Current itchy flexural rash	1.13 (0.87–1.47)	1.06 (0.65–1.75)	1.33 (0.98–1.82)	1.14 (0.92–1.40)	1.09 (0.69–1.71)	0.86 (0.50–1.49)	1.05 (0.63–1.74)
Severe eczema	0.94 (0.44–1.98)	1.79 (0.59–5.38)	**1.94 (1.02–3.71)**	1.06 (0.60–1.87)	1.17 (0.36–3.81)	1.03 (0.26–4.07)	1.62 (0.49–5.41)
Ever doctor-diagnosed eczema	**1.25 (1.03–1.50)**	0.68 (0.44–1.06)	1.20 (0.94–1.52)	0.96 (0.83–1.12)	0.96 (0.66–1.39)	1.08 (0.77–1.50)	1.21 (0.87–1.68)
Study-defined current eczema	1.18 (0.90–1.55)	1.04 (0.62–1.74)	**1.45 (1.07–1.20)**	1.13 (0.91–1.40)	1.09 (0.68–1.73)	0.81 (0.45–1.46)	0.98 (0.57–1.67)

*PR, Prevalence ratio; CI, Confidence interval*.

* *Adjusted for sex, age, breastfeeding, mode of birth, environmental tobacco smoke exposure, body mass index, maternal history of allergy, paternal history of allergy, maternal education, paternal education, number of older siblings, and number of younger siblings*.

† *Regression model failed to converge due to counts being zero in some cells. Hence, prevalence ratio was not estimated*.

*Boldface: P-value < 0.05*.

## Discussion

In this cross-sectional study, we explored pet-keeping patterns in families with children in Kuwait and investigated the relationship between exposure to different pets and symptoms of asthma, rhinitis, and eczema among adolescents. The 12-month prevalence of any pet ownership was estimated to be 42.8%, and birds (28.3%) were the most commonly kept pets. Participants who kept birds at home had a higher prevalence of study-defined current rhinitis and current itchy rash than those who did not keep birds. Moreover, rabbit-keeping was significantly associated with multiple symptoms of asthma and eczema. Also, cat-keeping showed associations with current wheezing, current rhinitis symptoms, and ever doctor-diagnosed eczema. Dog-keeping was not associated with current allergic symptoms in our study. The findings of this report demonstrate that pet-keeping is common and diverse in Kuwait, and keeping pets is associated with symptoms of allergic diseases among adolescents.

One initial objective of the current study was to estimate the prevalence of pet-keeping among families with children in Kuwait. Our results showed that 42.8% of the participating families had kept pets in their households in the past 12 months. The keeping of birds, cats, rabbits, fish, and dogs was reported by 28.3, 13.2, 7.8, 3.9, and 3.1% of households, respectively. Consistent with our findings, a study from Turkey reported that birds were the most commonly kept pets, followed by cats, fish, and dogs (31). A study from the United Arab Emirates (UAE) found the prevalence of any pet-keeping to be 40.7%, similar to our estimate of 42.8% (32). Moreover, the estimate of cat-keeping in our study (13.2%) was similar to that reported by the study conducted in the UAE (14.6%) (32). However, in contrast to our findings, studies based on western populations showed that cats and dogs were the most commonly kept pets (16, 33). For instance, the ISAAC estimated that 20 and 29% of households in countries in “Northern and Eastern Europe” kept cats and dogs, respectively; however, only 12 and 3% of households in countries in the “Eastern Mediterranean” region reported keeping cats and dogs, respectively (18). Such discrepancies in pet-keeping patterns may be attributed to several factors, including climatological and cultural/religious differences. In Kuwait and other gulf countries, the predominantly subtropical desert climate seems to influence people into staying indoors; hence, they may prefer to keep small pets that are easy to take care of and do not require outdoor spaces/activities. Another possibility is that families living in households with carpet flooring may favor caged pets (e.g., birds) over free-roaming furry pets. In addition, religious backgrounds may play a role in such practices; in fact, many Muslim scholars consider dog saliva ritually impure.

Most previous studies have assessed the associations of cat and dog exposure with allergic disorders, and the reported associations have been contradictory (16, 17, 34, 35). Our results showed that cat-keeping was associated with asthma and rhinitis symptoms, as well as ever doctor-diagnosed eczema. Such findings are consistent with results from the ISAAC investigation, which showed positive associations between cat exposure and allergic symptoms (18). Moreover, a Chinese study, conducted among children aged 0–8 years, showed that current cat ownership increased the risk of being diagnosed with asthma, rhinitis, and eczema (22). Similarly, a local case–control study from Kuwait reported an association between cat exposure and physician-diagnosed asthma among children aged 9–16 years (36).

The current study did not find any associations between dog exposure and symptoms of asthma, rhinitis, and eczema, although previous studies conducted in western countries have reported positive associations between dog exposure and allergic diseases (18, 33). Similar to our findings, a study conducted among preschool children in Shanghai, China did not find significant associations between current dog-keeping and symptoms of allergic diseases (37). Moreover, two studies from Japan reported no associations between dog-keeping and respiratory and allergic symptoms (38, 39). Dog-keeping was reported by 3.1% of households in the current study, corroborating previous findings of a lower frequency of dog ownership (1.5%) and a lower level of dog allergens (Can f 1) in Kuwait than in western countries, with the Can f-1 level being below the sensitization threshold in Kuwaiti households (36). Nevertheless, dog-keeping showed non-statistically significant trends for association with study-defined current asthma (aPR = 1.28, 95% CI 0.90–1.80), severe rhinoconjunctivitis (aPR = 1.66, 95% CI 0.42–6.56), and severe eczema symptoms (aPR = 1.79, 95% CI 0.59–5.38; Table 4). Such observations indicate that the low prevalence of dog-keeping (i.e., few number of families kept dogs) in our study sample could have led to not finding statistically significant effects of dog-keeping on allergic diseases.

Little is known about rabbit-related allergy in non-occupational environments. While extensive research on pet-keeping has been conducted, there have been very few reports on rabbit-induced allergy in domestic settings. Several rabbit allergens (e.g., Ory c 1, Ory c 2, Ory c 3, Ory c 4, and rabbit serum albumin) have been identified in hair, dander, saliva, and urine, and these may be found floating in the air or settling on different household surfaces (40–43). In this study, rabbit-keeping was positively associated with multiple symptoms of asthma and eczema. In Italy, a study conducted on patients with allergic rhinitis and/or asthma found that rabbit owners who were mono-sensitized to rabbit allergens experienced persistent respiratory symptoms (44). In addition, a report from Korea described three cases of rabbit-induced asthma and/or rhinitis resulting from domestic exposure to rabbits (36). To this end, our results of the positive association between rabbit exposure and symptoms of asthma and eczema are novel and should be corroborated. Given that rabbits are becoming very common as pets, ranked third after cats and dogs in Europe and the US (41), future prospective studies on the effect of rabbit exposure and allergen levels on the risk of allergic diseases are needed to achieve a better understanding of the impact of rabbit-keeping.

Birds were found to be the most commonly kept pet in the current study, and household bird-keeping was found to be positively associated with rhinitis and eczema symptoms. Such findings are consistent with those of previous studies. For example, a cross-sectional study from Qatar, conducted among children aged 6–14 years, showed that exposure to birds was significantly associated with allergic rhinitis and eczema prevalence (45). Another investigation found bird feathers to be common indoor aero-allergens whose presence was significantly associated with allergic sensitization and the severity of allergic rhinitis (46). Furthermore, a study conducted in Czech Republic found a positive relationship between the levels of bird feather allergens and the severity of eczema (47). Several prior investigations showed that bird feathers, serum, and droppings can carry several inhaled allergenic components and mites that may induce allergic responses in exposed individuals (42, 48–50). In addition, birds' cages *per se* have been identified as an environment with high levels of various airborne contaminants, including bird excreta, different types of bacteria and endotoxins, and aerosolized particles of food remnants (49, 51).

In our study, we also found that exposure to reptiles was associated with an increased prevalence of dry cough at night. Although this is rarely investigated, exposure to reptiles seems to be associated with adverse upper and lower respiratory tract symptoms (42).

The present study is the first to provide data on the prevalence of pet-keeping in Kuwait for different types of domestic animals. Another key strength of the current study is the large sample size and wide geographical coverage of all governorates of Kuwait, providing a well-representative sample of families with children in Kuwait. Moreover, the information on pet types/groups adds to the strengths of the current report. This study, however, has some limitations, the first of which is the absence of data on selective pet avoidance behavior, where subjects/families at high risk for allergies might tend to avoid keeping pets. However, we used maternal/paternal history of allergy as a surrogate marker for this behavior and investigated its association with pet-keeping (Table 3). In general, maternal/paternal history of allergy was associated with an increased prevalence of pet-keeping in our study. Such findings contradict the theory of selective avoidance and indicate that lack of awareness, possibly, led parents with allergy to not avoid pets. Thus, avoidance behavior may not have been a major confounder in our analysis. A recent analysis of ISAAC data, addressing the effect of reverse causation due to selective avoidance, showed that exposure to cats and dogs was a stronger risk factor for eczema and wheezing in non-affluent countries (where there is, perhaps, less avoidance/awareness) than in affluent countries (23, 24). Another arguable limitation of this cross-sectional study may be recall bias. Parents with allergy may tend to over-report pet-keeping and allergic symptoms when compared to families where individuals do not have allergy. Although no objective measures were used in this study to assess allergic status among participants, the subjective reported symptoms in the questionnaire were based on core questions from the ISAAC questionnaire. Another potential limitation to our study is that weight and height of the children were reported by the parents/guardians, which could have led to the misclassification of children into BMI-for-age groups. However, the estimated prevalence of overweight (25.4%) and obesity (28.8%) in our report did not substantially differ from estimates of a previous investigation conducted among schoolchildren aged 6–18 years in Kuwait that used objective weight and height measurements (overweight: 21.6% and obesity: 30.5%) (52). Moreover, it is essential to note that our analysis aimed to assess associations between different exposures and allergic symptoms, and not to infer causal relationships.

In conclusion, household pet-keeping is very common in Kuwait and pet-keeping behavior is diverse. Household pet-keeping was found to be positively associated with symptoms of allergic diseases. Birds were the most commonly kept pets among families with children in Kuwait, followed by cats and rabbits. Exposure to cats, rabbits, and birds showed associations with symptoms of asthma, rhinitis, and eczema among adolescents. Although rabbits are becoming very popular as pets, there are very few studies on their role in inducing allergic diseases. This study showed that rabbit-keeping is associated with multiple asthma and eczema symptoms, and this interesting observation warrants further corroboration.

## Data Availability Statement

The datasets generated for this study are available on request to the corresponding author.

## Ethics Statement

The studies involving human participants were reviewed and approved by The Standing Committee for Coordination of Health and Medical Research, Ministry of Health, Kuwait (no. 2016/451). Written informed consent to participate in this study was provided by the participants' legal guardian/next of kin.

## Author Contributions

KA contributed to the conception and design of the study, analyzed and interpreted the data, and drafted the manuscript. AZ conceived, designed, and planned the study, obtained funding, supervised the research conducted, analyzed and interpreted the data, and reviewed the manuscript. All authors critically revised the manuscript for important intellectual content. All authors contributed to the article and approved the submitted version.

## Conflict of Interest

The authors declare that the research was conducted in the absence of any commercial or financial relationships that could be construed as a potential conflict of interest.
