# Supplementary material for: Pet-Keeping in Relation to Asthma, Rhinitis, and Eczema Symptoms Among Adolescents in Kuwait: A Cross-Sectional Study
Source: Front Pediatr. 2020 Jun 23;8:331. doi: 10.3389/fped.2020.00331 (PMC7324793; doi:10.3389/fped.2020.00331)
Supplement: Supplementary file 1 [file Table_1.DOCX]

**Table S1.** Prevalence of household pet-keeping according to pet type

| **Pet type** | **% (n/total)** | **95% CI** |
| --- | --- | --- |
| Cat | 13.2 (506/3824) | 12.2-14.3 |
| Dog | 3.1 (120/3833) | 2.6-3.7 |
| Bird | 28.3 (1076/3809) | 26.8-29.7 |
| Rabbit | 7.8 (296/3809) | 6.9-8.6 |
| Poultry | 3.3 (124/3809) | 2.7-3.8 |
| Reptile | 3.8 (146/3809) | 3.2-4.4 |
| Fish | 3.9 (149/3809) | 3.3-4.5 |
| Rodent | 0.9 (34/3809) | 0.6-1.2 |
| Any pet | 42.8 (1643/3841) | 41.2-44.3 |
